# Lifestyle Interventions in an Aged Population: Challenges and Opportunities from a Public Health Perspective

**DOI:** 10.3390/nu16010173

**Published:** 2024-01-04

**Authors:** Naomi Cano-Ibáñez, Aurora Bueno-Cavanillas

**Affiliations:** 1 Department of Preventive Medicine and Public Health, University of Granada, 18071 Granada, Spain; abueno@ugr.es; 2 Consortium for Biomedical Research in Epidemiology and Public Health (CIBERESP), 28029 Madrid, Spain; 3 Instituto de Investigación Biosanitaria (ibs. GRANADA), Complejo Hospitales Universitarios de Granada/Universidad de Granada, 18071 Granada, Spain

## 1. Introduction

In recent decades, the prevalence of non-communicable diseases (NCDs) such as obesity, type 2 diabetes mellitus, cancer and cardiovascular disease has increased worldwide [1]. Although these conditions affect a cross-section of the population, the diseases are particularly substantial in older age groups [2]. Healthcare advances have increased life expectancy, but there is also an estimated gap of 280 million disability-adjusted life years globally [1]. This background has attracted interest in research for guiding actions based on elderly lifestyles. These interventions are crucial not only to prevent negative outcomes, but also to reduce polypharmacy, functional decline, and institutionalization [3,4], in order to decrease the impact over the health economy [5].

Among the main modifiable risk factors for the development of NCDs, diet plays a fundamental role in both prevention and control. Older adults are highly susceptible to diet misconceptions and are one of the main groups placed at high nutritional risk [6]. Increasing age is accompanied by a decrease in autonomy, economic capacity, and quality of life, and an increase in loneliness [7]. These factors are directly related to dietary choices and food variability, leading to unbalanced dietary intake [8]. Moreover, the ageing process is also associated with a higher prevalence of concomitant NCDs, drug abuse, and physiologic changes such as tooth loss and nutrient malabsorption [9]. These factors can intensify nutrient deficiencies that may aggravate NCDs and drive the onset of new diseases.

This Editorial introduces the Special Issue “Effect of Lifestyle and Diet for Older Persons’ Health”. The Special Issue highlights the current state of knowledge on public health issues related to the effects of lifestyle and diet on the health of older people. It contains fifteen articles and three reviews. These will be briefly outlined alongside the editorial to motivate readers to delve into their content.

## 2. A Summary of Published Articles

Frailty and physical disability in the elderly often manifests in the appearance of musculoskeletal conditions. One of the most studied diseases is sarcopenia, resulting in a loss of muscle strength and mass. While sarcopenia is frequently linked to oropharyngeal dysphagia, a retrospective study on 109 geriatric patients by Calles et al. (contribution 1) revealed no significant association. The authors concluded that muscle mass and function do not significantly contribute to the deterioration of swallowing and malnutrition in the geriatric population. Among dietary factors, amino acids, with nutritional and regulatory functions, play an essential role in muscle metabolism. Yi Su et al. (contribution 2) revealed that higher levels of circulating branched-chain amino acids (BCAA) and total glutathione (tGSH) were linked to healthier muscle status in older individuals. Conversely, a pro-inflammatory diet and elevated concentrations of sulfur-containing amino acids (SAAs), total homocysteine (tHcy), cystathionine, and total cystathionine (tCys) were associated with poorer muscle status over a 4-year period (*p* < 0.005). Similarly, Minqi Liaio et al. (contribution 3) showed, in a cohort of 4336 Chinese community-dwelling elderly people, that the odds ratio (OR) of muscle weakness decreased with BCAA consumption both in men and women (0.50 (0.38–0.65) and 0.67 (0.50–0.91), respectively), with low physical performance, both for 4 m walking speed (OR = 0.68; 0.50–0.93) and for repeated chair rises (OR = 0.66; 0.54–0.81).

Plant-based and animal-based proteins have been identified as key sources of amino acids (AA) in the human diet. A randomized controlled trial, carried out by de Marco Castro et al. (contribution 4), showed that animal-based protein effectively increases postprandial AA concentration. Nevertheless, factors like digestibility, fiber content, and the environmental impact of plant-based proteins should be considered when formulating a well-rounded diet for the elderly.

Another skeletal system disease widely studied in older people is osteoporosis. Inadequate nutrient intake adversely affects bone health, increasing the likelihood of falls and osteoporotic fractures. The review conducted by Kupisz-Urbanska (contribution 5) in this Special Issue showed that a multidisciplinary intervention based on screening for older adults at risk and a healthy balanced diet with a caloric intake of 30 kcal/kg/day and at least 1 g of protein per kg of body weight per day prevent the vicious cycle of falls, fractures, and nutrient scarcity. Both sarcopenia and osteoporosis are closely interrelated among the elderly. These problems increase with adiposity, resulting in osteosarcopenic adipose syndrome (OSA). Data from 365 residents in Croatian nursing homes were evaluated showing that intramuscular adipose tissue was significant and positively associated with OSA. Additionally, these patients exhibited a high prevalence of malnutrition, with rates of 5.9% in women and 10.0% in men (contribution 6).

Neurocognitive and mental disorders are more frequent in the elderly population. Exposure to risk factors such as dietary choices and physical inactivity throughout the life-cycle shows an accumulative risk over time. Some specific diet components have been directly related to a better control of ageing transition, attracting the attention of nutritional epidemiology. Among them is the adequate intake of omega-3 polyunsaturated fatty acids (n-3 PUFA) during menopause, which is purported to have a positive effect on emotional and cognitive functioning (contribution 7). Also, vitamin D has been linked to better cognitive status. Concerning to this micronutrient, a prospective multicenter-cohort study showed that subjects with vitamin D deficiency (<25 nmol/L) were at increased risk for all-cause dementia (Hazard Ratio (HR) = 1.91 (95% CI 1.30; 2.81)) and Alzheimer’s disease (HR = 2.28 (95% CI 1.47; 3.53)) (contribution 8). However, in nutritional epidemiology, it is of particular interest to evaluate the effect of overall dietary patterns instead of isolated nutrients. Each unit increase in the Mediterranean Diet Adherence score was associated with a 6.2% decrease in the risk for depression (*p* = 0.001), as reported by Eirini Mamalaki et al. (contribution 9) in the HELIAD Study, an observational research undertaking with a sample of 879 Greek community-dwelling older adults aged ≥65 years old. The hydration status of older adults with neurodegenerative diseases is crucial, as it is influenced by both physiological and social factors that affect fluid intake. More than 65% of the sample of “Body & Brain” study did not reach the reference values for water intake (contribution 10). A realistic strategy to increase water consumption in elderly is the daily inclusion of vegetables, fruits, or dairy products.

All of the aforementioned pathologies lead to an increase in the risk of mortality in the elderly. Some core strategies addressing the prevention of excess mortality have focused on diet quality. One of the indicators which measure the quality of dietary intake is dietetic diversity or variety. Torres-Collado et al. (contribution 11) compared participants with low vs. high diversity intake, reporting that participants with higher diversity had 32% and 45% less risk of death for all-cause and for cardiovascular disease, respectively, than lower diversity. Sodium and alcohol intake have been also related to an excess of mortality in older people. The results of the Prospective Lifelines-MINUTHE study emphasize the combined impact of sodium and protein intake on all-cause mortality. The group with low sodium intake and higher protein intake exhibited the lowest risk. In contrast, a low intake of sodium and protein was associated with an increased mortality risk, allegedly due to poor nutritional status (contribution 12). As mentioned earlier, maintaining an appropriate balance of dietary protein is crucial. However, it is noteworthy that protein-energy malnutrition is prevalent among the elderly. Some behavioral determinants such as knowledge and social support were independently associated with the chance of a low protein intake (contribution 13).

Other lifestyle habits like alcohol consumption remain controversial in nutritional epidemiology. Barbería-Latasa and cols. (contribution 14) analyzed the adherence to a Mediterranean Alcohol-Drinking Pattern (MADP) in the prospective SUN study. They found a lower risk of mortality among those with high adherence to the MADP than those with low adherence (OR = 0.54; 95% CI: 0.37–0.80). The messages to be conveyed regarding alcohol intake are: “*The pattern approach to the study of alcohol consumption should be more widely used for analyses than simply grams of alcohol consumed*”; for older people, “*If you drink alcohol, high adherence to the MADP score could substantially reduce your risk of all-cause mortality*”.

Primary preventive interventions for the elderly should emphasize lifestyle, but it is equally important for clinicians and researchers to focus on rehabilitation and prehabilitation. This involves prescribing appropriate dietary intake and physical activity before providing health assistance. Dennis van Erck and colleagues (contribution 15) found in their research that the majority of patients undergoing transcathether aortic valve implantation (TAVI) presented poor nutritional and physical activity status before and after the procedure. They emphasized the importance of counseling before and after TAVI surgery, focusing on low-intensity functional exercises and providing dietary advice on protein and whole grains for positive recovery. On the same lines, Yu-Ching Lin et al. (contribution 16) demonstrated in a cohort of prostatic adenocarcinoma patients that the combination of aerobic and resistance exercise prevents lean mass loss, decreases PSA levels, and avoids the need for nutrition with androgen deprivation therapy treatment in older men. Other chronic patients, such as those affected by age-related hearing disorders, can benefit from the promotion of a MedDiet pattern characterized by higher amounts of vitamins A, C, and E, which act as antioxidants, the regular practice of physical activity, fair sleep quality, smoking cessation, and the avoidance of noise exposure (contribution 17).

None of the aforementioned interventions can be applied if researchers do not identify the circumstances surrounding aging. The elderly have higher rates of low income, reduced appetite, and difficulties in obtaining and preparing food, leading to food insecurity and suboptimal nutritional status (contribution 18).

## 3. Issues, Challenges, and Limitations

Researchers and clinical practitioners should employ multidisciplinary approaches when investigating the process of healthy aging. However, the specific characteristics of this population group could make research difficult. There are many issues to consider. A controversial aspect is ethical issues. Research involving older adults may raise ethical questions related to informed consent, vulnerability, and privacy, particularly for non-community dwelling individuals who present with disabling diseases. Other aspects are directly related to the economics of longitudinal research on lifestyle factors, which can be costly and logistically challenging. Social inequalities could manifest themselves in low-resource settings, where life expectancy is generally lower than in high-income countries. Here, ageing would not be a funding research priority. Even in high-income countries, financial security in later life can impact the ability to critically evaluate and adopt a healthy lifestyle among older individuals. The implementation of preventive healthcare through specific vaccinations, health-related screening, cognitive stimulation, regular exercise and balanced diet, as well as considerations of elder abuse, is essential to maintain the health status of the elderly. However, these activities must be implemented cooperatively across governmental policies, ensuring financial security in retirement and providing adequate access to healthcare systems. Multifaceted and holistic strategies and policies need to focus on physical, mental, and emotional well-being for ensuring the safety of elders in the community.

## 4. Conclusions

The studies featured in this Special Issue influence health practices and policies, directly impacting life-long care. The adoption of a balanced diet like the MedDiet can provide sufficient protein, n-3 PUFA, and vitamin D, and maintain low sodium levels. The MedDiet together with physical activity, correct hydration status, and control of adiposity offers the best possibility of achieving and maintaining an optimal health status. This approach, valid at any age, is especially relevant in elderly subjects, contributing to the primary prevention of chronic diseases, and it also underpins the adequate control and prevention of chronic conditions and their complications.

## 5. Future Direction

The policies for the promotion of healthy lifestyle in the elderly must be translated into the healthcare system. The promotion of a healthy diet and the encouragement of physical activity should be addressed at all levels of care, particularly in primary care. Future directions must emphasize the identification of modifiable factors and barriers that may affect adherence to a healthy lifestyle throughout life. This would involve reshaping services and including professionals specially trained in nutritional counseling in the healthcare system. Economic evaluations are urgently needed to demonstrate the cost-effectiveness of these interventions. New interventions need evaluation for the prevention of falls and fractures. Lateral thinking may be needed. For example, anabolic steroids have been used in combination with exercise to improve muscle mass and strength in athletes; they may have similar effects in older people who experience sarcopenia. It is essential to provide training on creating a health-focused environment for all stakeholders, including researchers, guideline makers, charities, and lay readers. This training is crucial for prioritizing and guiding cost-effective interventions. Patient and public involvement would help staff to build knowledge around how to appropriately target lifestyle interventions in this vulnerable population [10].
